# Data on common variants associated with coronary artery disease/myocardial infarction in ethnic Arabs

**DOI:** 10.1016/j.dib.2016.02.010

**Published:** 2016-02-09

**Authors:** Salma M. Wakil, Ramesh Ram, Nzioka P. Muiya, Munish Mehta, Editha Andres, Nejat Mazhar, Batoul Baz, Samya Hagos, Maie Alshahid, Brian F. Meyer, Grant Morahan, Nduna Dzimiri

**Affiliations:** a Genetics Department, King Faisal Specialist Hospital and Research Centre, Riyadh, KSA, Saudi Arabia; b Harry Perkins Institute of Medical Research, University of Western Australia, Australia; c King Faisal Heart Institute, King Faisal Specialist Hospital and Research Centre, Riyadh, KSA, Saudi Arabia

## Abstract

The data shows results acquired in a large cohort of 5668 ethnic Arabs involved in a common variants association study for coronary artery disease (CAD) and myocardial infarction (MI) using the Affymetrix Axiom Genotyping platform (“A genome-wide association study reveals susceptibility loci for myocardial infarction/coronary artery disease in Saudi Arabs” Wakil et al. (2015) [1] ). Several loci were described that conferred risk for CAD or MI, some of which were validated in an independent set of samples. Principal Component (PCA) analysis suggested that the Saudi Cohort was close to the CEU and TSI populations, thus pointing to similarity with European populations.

**Specifications table**

**Table:** 

Subject area	*Genetics*
More specific subject area	*Genetics of complex cardiovascular diseases*
Type of data	*Tables and figures*
How data was acquired	*Data table was acquired using statistical methods by SPSS,*
Data format	*Raw and analyzed data*
Experimental factors	*None*
Experimental features	*Genome-wide association experiments were performed using Affymetrix platform; analysis performed using PLINK, GTCA, FASTLMM and principal Component Analysis*
Data source location	*All regions of Saudi Arabia*
Data accessibility	*Data is with this article*

**Value of the data**

• Genomic distribution of risk variants for CAD/MI in ethnic Arabs.
• Comparative analysis of the genomic distribution of the associated loci for CAD/MI between the Arab population and other ethnic groups.
• Regional association plots demonstrate loci on 2q33, 8q13, 9p31 for CAD and on 21q22.11 for MI.
• Principal component analysis comparison with 11 other MApMAp3 populations shows variations and clustering of ethnic populations.
• The Saudi Arab cohort show similarities with the Caucasian populations.

## 1 Data

**Fig. 1:**
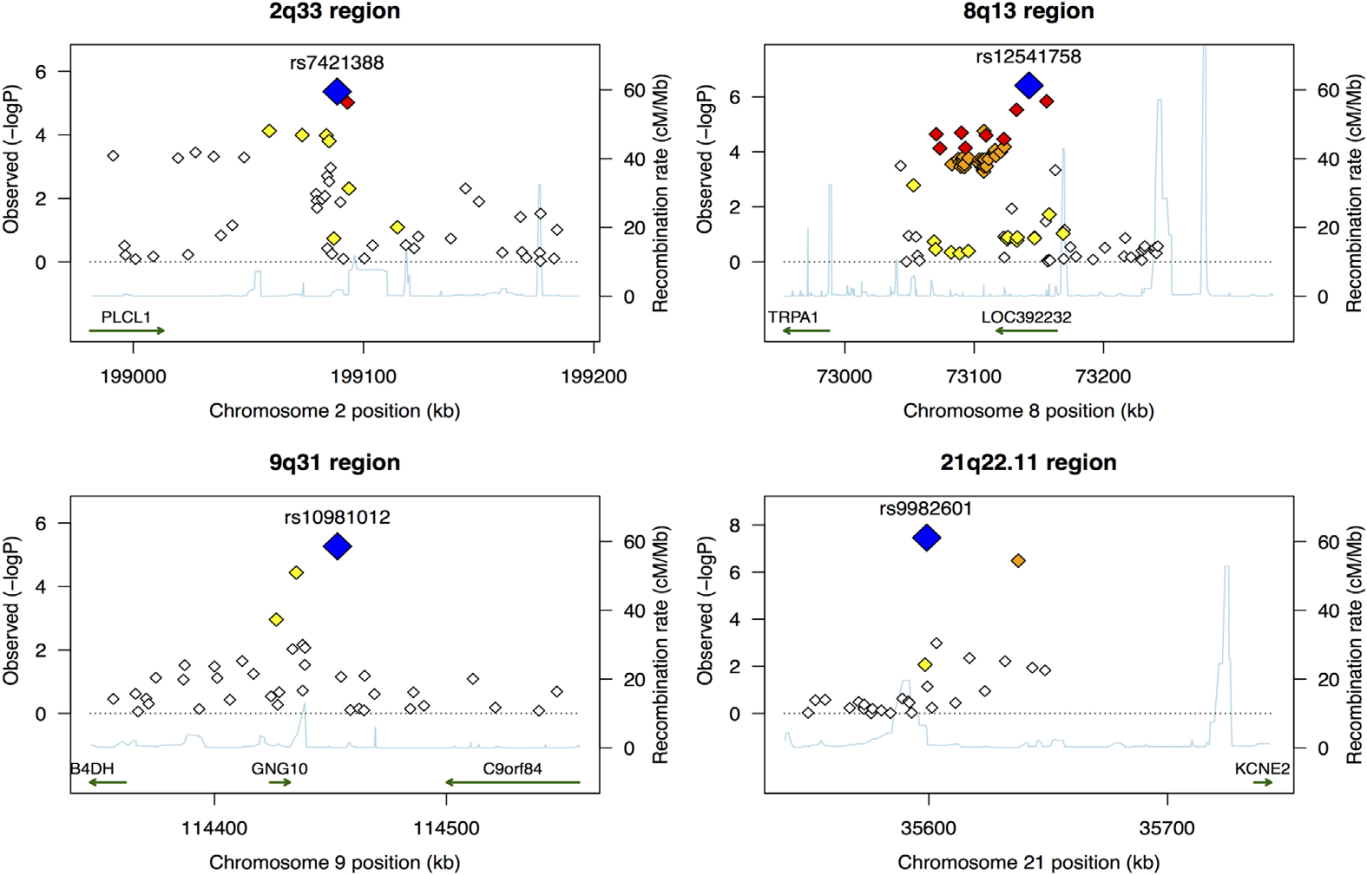
Regional association plots for coronary artery disease and myocardial infarction.

**Fig. 2:**
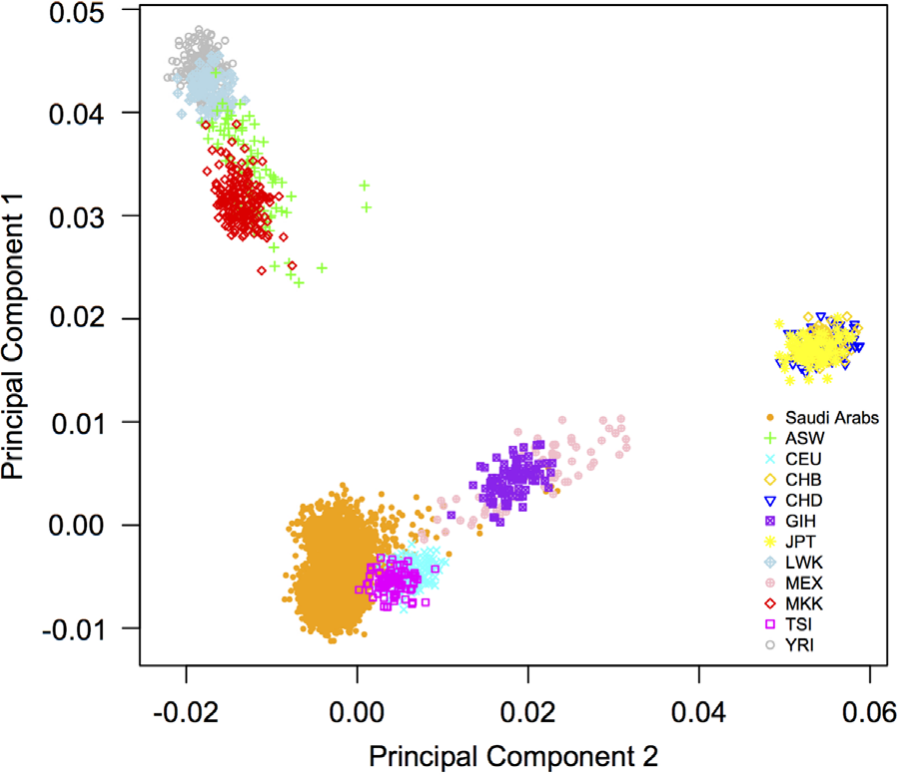
Principal Component Analysis.

**Fig. 3:**
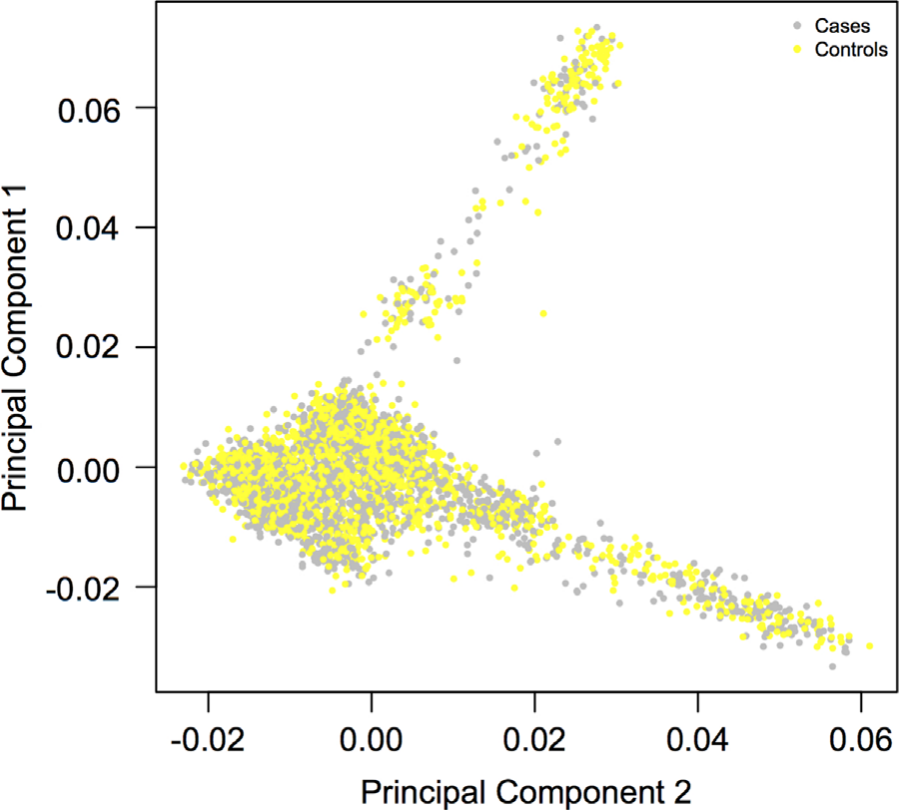
Principal component analysis.

The summary puts together the clinical features of the studied cases versus controls, the genomic distribution of the implicated variants and the principal component analysis of the data, as well as comparison of the Saudi population with other ethnic groups(Fig. 1, Fig. 2, Fig. 3)(Table 1).

The figure shows the loci on loci 2q33, 8q13, 9p31 associated with coronary artery disease and locus 21q22.11.associated with and myocardial infarction.

The figure shows the first and second principal component plot for the Saudi Arab samples (4431 samples: 2165 cases and 2266 controls) with eleven other HapMap3 populations (ASW, African ancestry in Southwest USA; CEU, Utah residents of European ancestry; CHB, Han Chinese in Beijing, China; CHD, Chinese in Metropolitan Denver, Colorado; JPT, Japanese in Tokyo, Japan; GIH, Gujarati Indians in Houston, Texas; LWK, Luhya in Webuye, Kenya; MKK, Maasai in Kinyawa, Kenya; TSI, Tuscans in Italy; YRI, Yoruba in Ibadan, Nigeria; MEX, Mexicans).

The figure displays the first and second principal component plot of the Saudi Arab samples (4431 samples: 2165 cases and 2266 controls) without Hapmap3 populations.

## 2 Experimental design, materials and methods

The discovery study involved 5668 Saudi Arabs who were subjected to genotyping using Affymetrix Axiom Genome-Wide ASI Array (Asian population). Genotyping data were generated using the Axiom GT1 algorithm and an IBS/IBD analysis in PLINK [2]. Analyses of the genome-wide association (GWA) were based on a linear mixed model method using FASTLMM-Select with Principal Component (PCs) as in Lippert et al. [3] and Widmer et al. [4]. Heritability estimation was performed according to Yang et al. [5] implemented in Genome-wide Complex Trait analysis (GCTA) software and extended in REACTA. The population substructure was examined by Principal Component Analysis (PCA) using the GCTA as described by Yang and colleagues [5] to eliminate the outliers that do not conform to the main cluster of samples that form the Saudi cohort, and may therefore lead to false positive results.

## Figures and Tables

**Table 1:** Important clinical features and demographics of the coronary artery disease cases (CAD) versus angiographed controls

	**CAD controls**			**CAD cases**		
	**All**	**Male**	**Female**	**All**	**Male**	**Female**
**Gender**	3000	1590(0.53)	1410(0.47)	2668	2028(0.76)	640(0.24)
**Age**	49.8±0.30	50.3±0.40	49.2±0.40	59.8±0.20	59.3±0.25	61.6±0.40
**BMI**	29.4±0.12	28.3±0.15	30.6±0.19	29.3±0.10	28.5±0.10	31.9±0.23
**MI**	933	589(0.63)	344(0.37)	2495	1919(0.77)	576(0.23)
**T2DM**	1207	646(0.54)	561(0.46)	1848	1354(0.73)	494(0.27)
**HTN**	1846	963(0.52)	883(0.48)	2187	1631(0.75)	556(0.25)
**lHDLC**	907	592(0.65)	315(0.35)	1353	1115(0.82)	238(0.18)
**hLDLC**	312	166(0.53)	146(0.47)	346	251(0.73)	95(0.27)
**hTG**	1846	963(0.52)	883(0.48)	2187	1631(0.75)	556(0.25)
**hChol**	710	365(0.51)	345(0.49)	1177	876(0.74)	301(0.26)
**FH**	679	378(0.56)	301(0.44)	500	397(0.79)	103(0.21)
**OBS**	1228	533(0.43)	695(0.57)	1087	710(0.65)	377(0.35)
**Smokers**	882	820(0.93)	62(0.07)	1251	1219(0.97)	32(0.03)
**VD**						
One	0	0	0	973	718(0.74)	255(0.26)
Two	0	0	0	529	408(0.77)	121(0.23)
>Two	0	0	0	1164	910(0.78)	254(0.22)

The numbers in brackets give the percentages of the total (all) values of the group. BMI, body mass index; FH, family history of CAD; MI, myocardial infarction; hLDLC, high low density lipoprotein-cholesterol level; lHDLC, low high density lipoprotein-cholesterol level; hTG, hypertriglyceridaemia; hChol, hypercholesterolaemia; HTN, hypertension; T2DM, type 2 diabetes mellitus; VD, number of diseased vessels.
