# Supplementary material for: Data on common variants associated with coronary artery disease/myocardial infarction in ethnic Arabs
Source: Data Brief. 2016 Feb 9;7:172–6. doi: 10.1016/j.dib.2016.02.010 (PMC5063810; doi:10.1016/j.dib.2016.02.010)
Supplement: Supplementary file 1 — Supplementary material [file mmc1.docx]

**Conflict of interest**

All authors declare that there is no conflict of interest to disclose.
